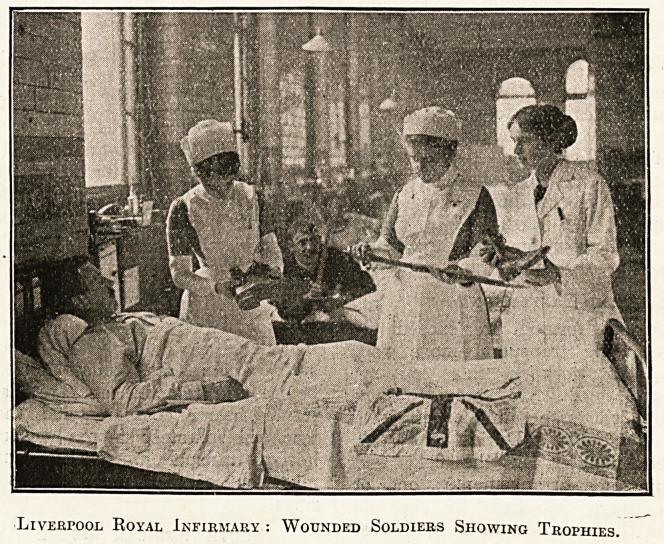# Liverpool Royal Infirmary and the Wounded

**Published:** 1915-03-13

**Authors:** 


### LIVERPOOL ROYAL INFIRMARY AND THE WOUNDED.

At the Liverpool Royal Infirmary two wards containing fifty beds have been placed at the disposal of the naval and military authorities. Since October 18 they have been very fully occupied, no fewer than 230 wounded having been received up to the present time. The injuries of most of these are to the feet and legs, and have not been of great severity, as such cases are necessarily dealt with nearer the port of arrival.

Liverpool Royal Infirmary: Wounded Soldiers Showing Trophies.

The infirmary, it should also be remembered, has been under promise to the authorities for some time to provide a certain number of trained nurses in case of national emergency, and on the outbreak of the war the majority of these were at once requisitioned for both Services. Sixteen have already gone, eight more may have to leave at a moment's notice, and four sisters have been appointed to the Liverpool Merchants' Mobile Hospital. The majority of the honorary staff are members of the Territorial section of the R.A.M.C., and are tending the wounded and invalided soldiers at the local base hospital at Fazakerley, while two have left for service abroad, with the two registrars and five of the house staff. Here as elsewhere the forms demanded by the military authorities are causing a large amount of clerical work.

## Figures and Tables

**Figure f1:**